# Slithering CSF: Cerebrospinal Fluid Dynamics in the Stationary and Moving Viper Boa, *Candoia aspera*

**DOI:** 10.3390/biology10070672

**Published:** 2021-07-16

**Authors:** Bruce A. Young, Skye Greer, Michael Cramberg

**Affiliations:** Department of Anatomy, Kirksville College of Osteopathic Medicine, Kirksville, MO 63501, USA; sa204910@atsu.edu (S.G.); mcramberg@atsu.edu (M.C.)

**Keywords:** reptilia, locomotion, fluid mechanics, spine, movement

## Abstract

**Simple Summary:**

The cerebrospinal fluid (CSF) flows through and around the central nervous system to nourish, cleanse, and support the brain and spinal cord. Though abnormalities of this CSF flow have been linked to multiple human neural diseases, little is known about the underlying mechanics of CSF flow. This study was designed to test the hypothesis that movement of the body’s trunk could cause CSF flow; hence, the study was conducted on a snake, an animal with prominent trunk movement. The results demonstrate that the resting snake has a CSF pressure profile that is very similar to what is seen in humans and other mammals, and that the CSF dynamics are changed during either artificial (manual) or natural (locomotor) movement of the snake’s body

**Abstract:**

In the viper boa (*Candoia aspera*), the cerebrospinal fluid (CSF) shows two stable overlapping patterns of pulsations: low-frequency (0.08 Hz) pulses with a mean amplitude of 4.1 mmHg that correspond to the ventilatory cycle, and higher-frequency (0.66 Hz) pulses with a mean amplitude of 1.2 mmHg that correspond to the cardiac cycle. Manual oscillations of anesthetized *C. aspera* induced propagating sinusoidal body waves. These waves resulted in a different pattern of CSF pulsations with frequencies corresponding to the displacement frequency of the body and with amplitudes greater than those of the cardiac or ventilatory cycles. After recovery from anesthesia, the snakes moved independently using lateral undulation and concertina locomotion. The episodes of lateral undulation produced similar influences on the CSF pressure as were observed during the manual oscillations, though the induced CSF pulsations were of lower amplitude during lateral undulation. No impact on the CSF was found while *C. aspera* was performing concertina locomotion. The relationship between the propagation of the body and the CSF pulsations suggests that the body movements produce an impulse on the spinal CSF.

## 1. Introduction

Perspectives on the cerebrospinal fluid (CSF) have changed; what once was seen as an almost “passive” supporting substance is now generally seen as a dynamic system, perturbations of which can cause a number of neural disorders [1,2]. Despite the increased recognition of the importance of the CSF, many aspects of the underlying fluid mechanics have not been elucidated (for a review see [3]). In humans and other mammals, the cardiac cycle and ensuing arterial pulsations have the greatest influence on CSF pulsations [4], while the ventilatory cycle has the greatest influence on CSF flow [5]. A recent study on the American alligator (*Alligator mississippiensis*) revealed that this mammalian pattern is not universal; in the alligator, there are both cardiac and ventilatory influences on the CSF, but they are far more variable than what has been reported from any mammal [6]. The basis for this variation is unknown; compared to mammals, both the cardiac output [7,8] and ventilatory cycle [9,10] of the alligator are actively variable.

Relating the alligator and mammalian CSF dynamics is challenging not only because of the greater plasticity of the alligator systems, but also because of the limits of our understanding of the underlying mechanics of CSF flow. Multiple studies have documented the relationship between the ventilatory cycle and CSF flow in mammals [11,12,13], but the mechanistic basis for CSF propulsion has not been established [14]. Researchers have postulated that thoracic displacement during the ventilatory cycle directly deforms the dural sheath around the spinal cord [15] and that intrathoracic pressure changes during ventilation alter central venous pressure and, thereby, the CSF pressure [16]. However, experimental results have challenged both of these hypotheses [17,18]. Alligators have a diaphragm capable of sustaining transdiaphragmatic pressures [19], and can ventilate through costal displacement [20], but little is known about how the variation in intrathoracic pressure in *Alligator* influences central venous pressure or CSF flow dynamics.

While the cardiac and ventilatory cycles may have the greatest effect on CSF dynamics, other influences have been documented. Postural shifts create orthostatic gradients that influence CSF pressure in humans [21], other mammals [22], and alligators [23,24]. Coordinated beating of the ependymal cells’ apical cilia can create CSF flow [25]. Recent experimental work showed that contraction of the myodural bridge, a localized insertion of suboccipital muscles onto the spinal dura [26,27], causes localized changes in CSF pressure [28]. A more substantial relationship between skeletal muscle contraction and large-scale CSF flow has been shown in embryonic zebrafish; lateral deformation of the body (as during oscillatory swimming) produces flow of spinal CSF [29]. The mechanistic basis behind this CSF flow in the zebrafish has not been clarified. Is there a direct connection, such as an elaborate myodural bridge, between the axial musculature and the spinal dural sheath? If the embryos are laying on their side, is the deflected tail creating an orthostatic gradient? Are the intervertebral joints pliant enough in these embryonic fish to cause compression of the dural sheath? Can the rapid displacement of the fish’s tail generate an impulse, changing the momentum of the CSF?

Insight into these issues of CSF propulsion might be gained through an examination of CSF flow dynamics in snakes (Order Serpentes). Snakes have some of the same cardiac specializations that occur in crocodilians [30,31], though cardiac outflow is not as dynamic, or actively regulated, in snakes. Unlike crocodilians, snakes lack a diaphragm [32]; though they share with crocodilians a rather plastic ventilatory cycle and a tolerance for apnea [33,34]. Previous studies have examined the influence of orthostatic gradients on blood flow in snakes [35,36], and have found that terrestrial species, unlike arboreal forms, generally lack compensation for orthostatic pressures. Snakes, being secondarily limbless [37], locomote primarily through axial deflection [38]. A snake undulating horizontally would not produce an orthostatic gradient, but it would produce a propagating wave of intervertebral joint displacements [39]. As such, the moving snake provides an opportunity to explore the relationship between locomotor mechanics and CSF dynamics.

The purpose of this study is to explore the CSF dynamics in the viper boa, *Candoia aspera*. This is a terrestrial species [40] and so presumably has little specialization for tolerating orthostatic gradients [41]. *Candoia aspera* is a relatively heavy-bodied species [42] and so would be expected to locomote using primarily lateral undulation and concertina locomotion [43]. Among other differences between these two modes of locomotion, lateral undulation involves propagating waves of axial deflection and is generally faster, while concertina locomotion lacks propagating waves and is slower [44]. Herein, we will examine the CSF dynamics of *C. aspera*, particularly as they relate to the axial deflection typical of serpentine locomotion.

## 2. Materials and Methods

### 2.1. Live Animals

Seven adult specimens (snout-vent lengths of 60–102 cm, mass of 182–460 g) of *Candoia aspera* were obtained commercially. The snakes were housed in (60 × 120 × 35 cm) terraria within a special reptile-holding facility with a 12:12 hour light cycle, water ad libitum, and a temperature range of 28 to 32 °C. The animals were maintained on a diet of live and/or previously frozen rodents (*C. aspera* predominantly eats lizards in wild; keepers/dealers train them to eat rodents for convenience). 

### 2.2. Cardiac and Ventilatory Influence

The snakes were placed (individually) in an induction chamber and exposed to isoflurane until their righting reflex was extinguished and they no longer responded to tactile stimulation. The snakes were removed from the induction chamber, given an analgesic (Maloxicam at 0.2 mg/kg IM), then intubated. The snake was then placed on a stiff board (244 × 28 × 3.8 cm thick). The endotracheal tube was connected to a custom anesthesia system that included a ventilator pump (Harvard Apparatus; Holliston, MA, USA), Vaporstick anesthesia machine (Surgivet; Waukesha, WI, USA), isoflurane vaporizer (Surgivet), and Capnomac Ultima respiratory gas monitor (Datex-Engstrom; Tewsksbury, MA, USA). The snakes were maintained on a ventilatory pattern of 5 breaths per minute; the tidal volume, pressure, and peripheral resistance of the anesthesia machine were adjusted for each snake so that the airflow entering the snake’s respiratory system was just enough to cause body movement over the vascular lung. Once it was determined that a surgical plane of anesthesia was established, the isoflurane was reduced to 1.5–2%.

The snakes’ EKGs were recorded using two silver chloride surface cup electrodes (019-477200, GRASS, Natus Medical, Pleasanton, CA, USA), coated with a layer of conducting gel (Signagel, Parker Laboratories, Fairfield, NJ, USA). The electrodes were placed on the lateral surface of the snake on either side of the heart. The electrodes were connected to a P511 preamplifier (GRASS). A stainless steel surgical burr was used to bore an approximately 1.5 mm-diameter portal through the skull, slightly parasagittal and just caudal to the parietal–frontal suture. In snakes, as in all terrestrial vertebrates, the outer surface of the brain is bathed in CSF, which is isolated from the skull by the dura matter. Pre-surgical dissections indicated that at the location of the surgical portal through the skull, the dura was further away from the surface of the brain, resulting in a localized increase in the volume of CSF (a cistern). The surgical portal allowed for direct exposure of the dura mater surrounding this cistern; a small incision in the dura was used to inset a segment of PE tubing into the CSF-filled subarachnoid space. The PE tubing was connected to a P23AA fluid pressure transducer (Statham Gould; Cleveland, OH, USA), both of which were filled with reptilian Ringer’s solution. The pressure transducer was mounted to the board at a fixed site immediately adjacent to, and level with, the snake’s head, so that rotation of the snake did not produce a pressure head between the PE tubing and the transducer. The implanted PE tubing was snug, and the attachment was further secured by applying epoxy cement to the PE tubing and surrounding bone. The pressure transducer was coupled to a P122 preamplifier (GRASS). 

The CSF pressure, EKG, and ventilatory pattern (the exhalatory CO2 trace from the gas analyzer) were recorded simultaneously (at 2 kHz) using the MiDas data acquisition system (Xcitex; Woburn, MA, USA). Ninety second data records were gathered while the snake was anesthetized and being ventilated at 5 breaths/min; at least one recording per snake was taken in which the ventilator was turned off, forcing the snake into apnea. The CSF pressure transducers were individually calibrated following each experiment.

### 2.3. Orthostatic Gradients

The board holding the snake was anchored to a rotating spindle machined to have, in addition to a stable horizontal stop, fixed “stops” at 30° above and below the horizon. The snake’s head and tail were taped to the board so that the snake did not shift relative to the board, then 90 s rotation trials were performed. Each trial consisted of a 30 s horizontal baseline, a 30 s period in the rotated posture, then a 30 s return phase. To avoid artifacts (vibration in the board), none of the “transitional” periods were analyzed. 

### 2.4. Axial Deflection

Following the orthostatic trials, the EKG leads and the tape holding the snake’s tail to the board were removed. Hypo-allergenic fingernail polish was used to place visible markers at intervals (approximately every 5 cm) along the dorsal midline of the snake. A digital camera (DSC-RX 100M4, SONY; San Diego, CA, USA) recording at 480 fps was positioned over the snake. The snake was still anesthetized, and thus had little body tone; by manually wiggling the tail back-and-forth, it was possible to create oscillatory waves that propagated along the snake’s body (to the fixed head). Care was taken not to elevate the tail or any portion of the snake’s body. An LED flash was used to synchronize the video and CSF pressure recordings. 

Following these axial deflection trials, the snake was allowed to fully recover from the anesthesia, with the CSF pressure catheter still in place. When the snake was fully recovered from the anesthesia (gauged by the presence of tongue flicks, avoidance response to tactile stimulation, and voluntary locomotion) the snake was placed in a 120 × 47 cm filming cage which was lined with a soft material (designed to minimize frictional contact points). Synchronized high-speed digital video and CSF pressure recordings were made while the snake was locomoting (unrestrained). All video records were exported to motion analysis software (Kinovea; open sourced from: https://www.kinovea.org/download.html, accessed on 22 May 2021), which was used to quantify the kinematics of the marker points on the snake’s body. For statistical comparison, the kinematic traces and CSF pulses were rectified and normalized. Kinematic and CSF traces were imported into Spectra-Plus (Pioneer Hill Software; Poulsbo, WA, USA) for FFT and Power Spectrum analysis.

## 3. Results

### 3.1. Heart Rate

The seven specimens of *Candoia aspera* had a mean resting (anesthetized) heart rate of 40.1 beats per minute (bpm), with a standard deviation of 8.9. The standard deviation reflects that each snake had a distinct heart rate, with means ranging from 25.3 to 49.3 bpm. The specimens ranged in mass from 182 to 460 g; linear regression revealed that heart rate decreased by 0.5 bpm for every 10 g of snake body mass.

When the snakes were rotated 30° head-up, the mean heart rate decreased by 0.6% (s.d. = 4.4%); this decrease changed the mean heart rate from 40.1 to 39.86 bpm. Rotating the snakes to 30° head-down increased the mean heart rate by 0.04% (s.d. = 3.9%); this increase changed the mean heart rate from 40.1 to 40.12 bpm. A paired *t*-test found no significant difference (*t* = 0.457, *p* = 0.325, *n* = 18) in the changes in heart rate resulting from head-up or head-down rotations.

### 3.2. CSF in the Immobile Snake

**Figure 1:**
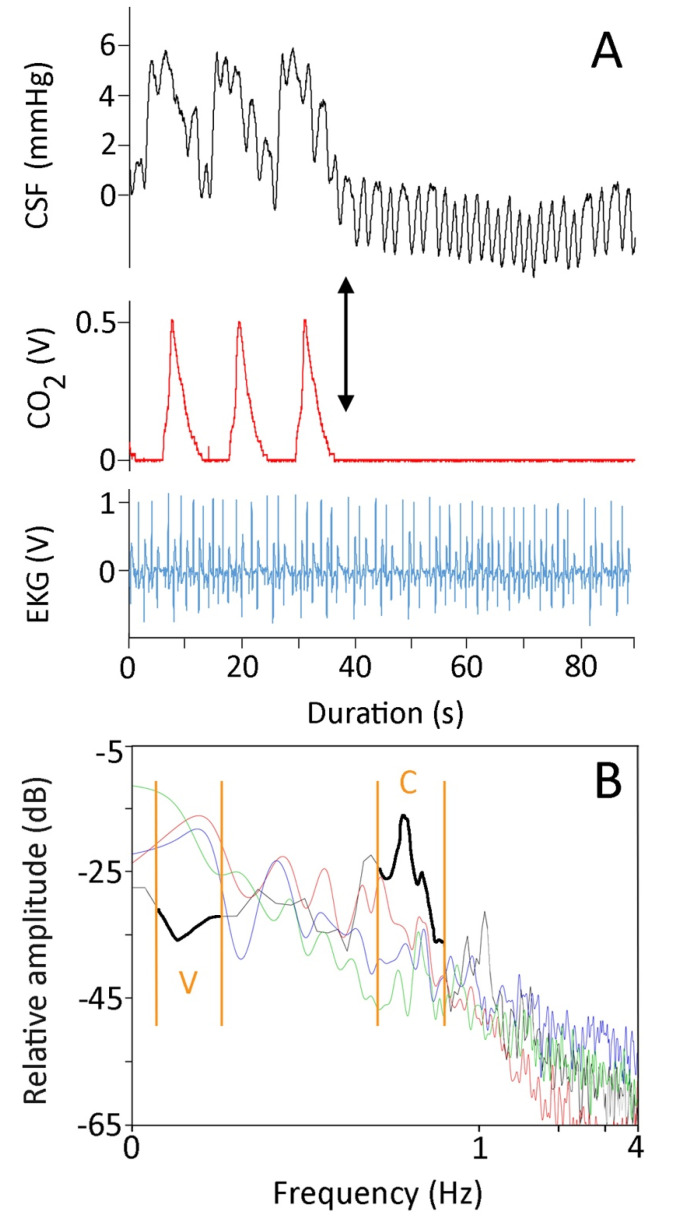
(**A**) For 90 s, simultaneously recorded CSF pressure (black, upper trace), exhalatory CO_2_ (red, middle trace), and EKG (blue, lower trace) from *Candoia aspera*. The left portion of the CSF trace exhibits low-frequency pulses which correspond to the ventilator cycle, and high-frequency pulses which correspond to the cardiac cycle. After three ventilator cycles, the ventilator was stopped (black arrow), forcing the snake into apnea and eliminating the low-frequency pulses from the CSF trace. (**B**) Power spectral analysis of four 90 s traces recorded from four different specimens of *Candoia aspera*. Three of the specimens (red, green, and blue traces) were ventilating at approximately the same rate and had similar heart rates. The data from the fourth specimen (black trace, highlighted for emphasis) were recorded while the specimen was in apnea; note that under apnea the power of the ventilatory component was lower while that of the cardiac component was higher. The cardiac (C) and ventilatory (V) ranges are denoted by the orange vertical lines.

The CSF had a mean resting pressure of 4.2 mmHg (s.e. = 0.3). In *Candoia*, there were two different frequencies evident in the pressure pulsation of the CSF (Figure 1A). The higher frequency pressure pulsations were temporally correlated to the EKG signals. Pooling 10 sampled pulsations from each specimen (all while the snake was anesthetized and maintained in a horizontal posture) revealed these higher frequency pulsations to have a mean amplitude of 1.2 mmHg (s.e. = 0.04). ANOVA found no significant differences among the amplitudes of these higher-frequency CSF pressure pulsations (F = 1.68, *p* = 0.14, df = 6).

The lower-frequency pressure pulsations were temporally correlated to the ventilator cycle, and disappeared during periods of induced apnea (Figure 1A). Pooling five of these pulses from each snake revealed these low-frequency pulsations to have a mean amplitude of 4.1 mmHg (s.e. = 0.09). ANOVA found significant differences among the amplitudes of these lower frequency CSF pressure pulsations (F = 85.92, *p* = 1.11 × 10^−16^, df = 6). Linear regression demonstrated that the amplitude of the ventilator-related pulses decreased with increasing body size (b = −0.03, R^2^ = 0.80). The low-frequency pulses (which occur at approximately 0.08 Hz) had an amplitude that was some 3.5× greater than those of the higher-frequency pulses (which occurred at approximately 0.66 Hz).

Power spectrum analyses of 90 s recordings of CSF pressure from horizontal anesthetized snakes showed a similar pattern; the frequencies associated with the ventilator cycle consistently had greater power than the frequencies associated with the cardiac cycle (Figure 1B). If the ventilator was turned off, so the snake was forced into apnea, the resulting power spectrum was dominated by the frequencies associated with the cardiac cycle (Figure 1B). 

**Figure 2:**
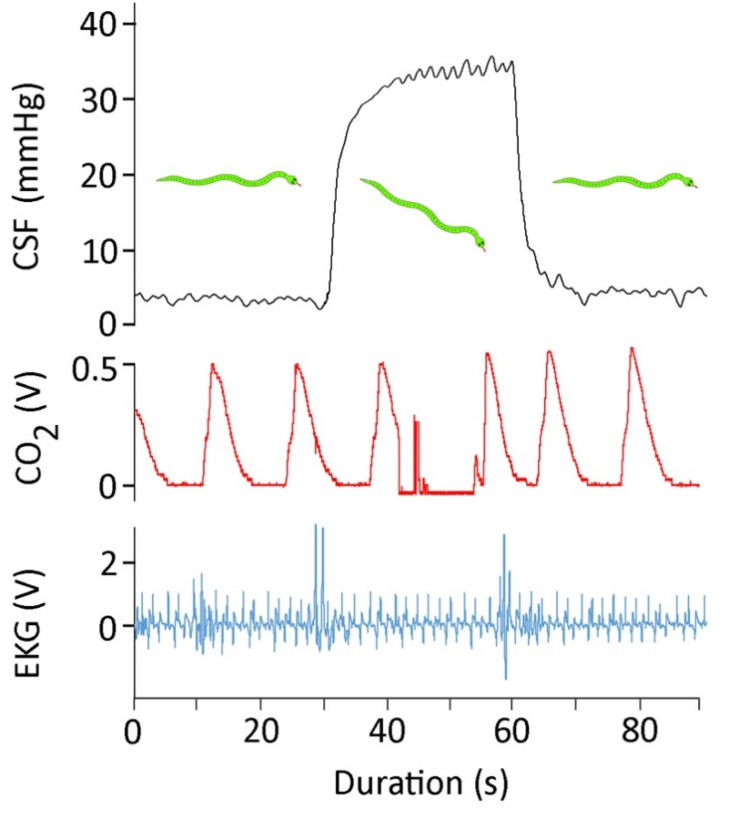
For 90 s, simultaneously recorded CSF pressure (black, upper trace), exhalatory CO_2_ (red, middle trace), and EKG (blue, lower trace) from *Candoia aspera*. This 90 s trace includes approximately 30 s of horizontal posture; then, the snake was rotated 30° head-down and held for 30 s, and finally the snake was returned to the horizontal.

The snakes were rotated either 30° head-up or 30° head-down in order to create an orthostatic gradient. The rotational trials produced three consistent results: (1) the CSF pressure changed rapidly upon rotation, and then held stable until the snake was returned to horizontal (Figure 2); (2) the increases in CSF pressure caused by head-down rotation were significantly larger in magnitude (*t* = 5.88, *p* < 0.00001, df = 19) than were the decreases in CSF pressure caused by head-up rotation; and (3) the amplitude of the high-frequency CSF pulses increased following head-down rotation, and decreased following head-up rotation (Figure 2). The 30° head-down rotations resulted in a mean increase in CSF pressure of 32 mmHg (s.e. = 1.51), while the 30° head-up rotations resulted in a mean decrease in CSF pressure of 17.6 mmHg (s.e. = 2.2).

### 3.3. Manual Axial Deflection

**Figure 3:**
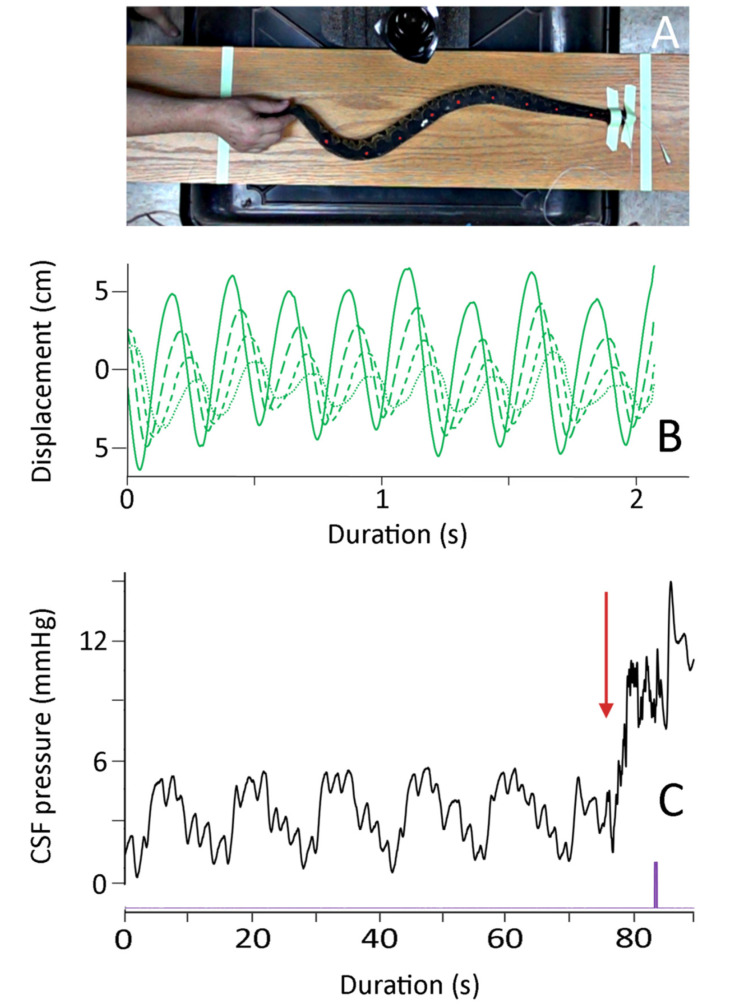
Summary of the manual oscillations. (**A**) Isolated frame from the high-speed digital video; note that the snake’s body is not elevated, the head is secured, and the marker points on the snake’s dorsal surface are clearly evident. (**B**) Kinematic data showing the lateral displacement of four successive body marker points (successive points have finer line dashes); (**C**) Cranial CSF pressure recorded during a manual oscillation trial. Note that the clear pattern of ventilatory (V) and cardiac (**C**) cycles in the CSF pressure is disrupted at the onset of manual oscillations (red arrow). The manual oscillations produced a rapid increase in the baseline CSF pressure, as well as higher-frequency pulsations during the oscillation trials. The purple marker is an integration signal that appeared in all kinematic and pressure records, allowing for the temporal alignment of the data sets.

The manual axial deflections were performed as the snake was recovering from anesthesia, but prior to the snake developing muscle tone. The EKG leads were removed, and to preserve the integrity of the CSF pressure catheter the snake’s head was taped to the board (those forming a stationary node for the undulations). The endotracheal tube was left in place, and was connected to the ventilator during some of the trials; in other trials (where the snake exhibited more signs of recovery), the endotracheal tube was not connected to the ventilator. By moving the snake’s tail rapidly horizontally it was possible to produce a series of lateral undulations which propagated towards the snake’s head (Figure 3). Pooling three trials from each of the seven snakes yields the kinematic profile of these manual oscillations given in Table 1. 

These manual oscillations were not intended to duplicate locomotion; rather, the goal was to induce vertebral deflection and rapid but realistic changes in direction (Figure 3). Every trial had multiple changes in directions (mean of 7.7 oscillations, and a mean oscillation frequency of 2.9 Hz). Both the oscillation velocity and the propagation velocities were approximately 1–2 body lengths per second. Note that the values given in Table 1 are not adjusted for the snake’s body length.

As shown in Figure 3, the onset of the manual oscillations was always associated with an increase in cranial CSF pressure. For the 21 trials, the CSF pressure baseline increased by a mean of 8.4 mm Hg (s.e. 0.18) relative to the pre-oscillation baseline. Once increased, this baseline did not return to the resting (pre-oscillation) level during the manual oscillations. The experimental protocol did not include the analysis of return of the CSF pressures to their resting levels. The elevation of the CSF pressure baseline was compared to the kinematic variables given in Table 1. Only one kinematic variable had a significant relationship with the magnitude of the CSF pressure baseline increase; increasing propagation velocity of the snake’s body undulations resulted in greater increases in CSF baseline pressure (b = 0.021, R^2^ = 0.48; this slope was significantly different from zero, *t* = 4.15, *p* = 0.0005, df = 19).

**Figure 4:**
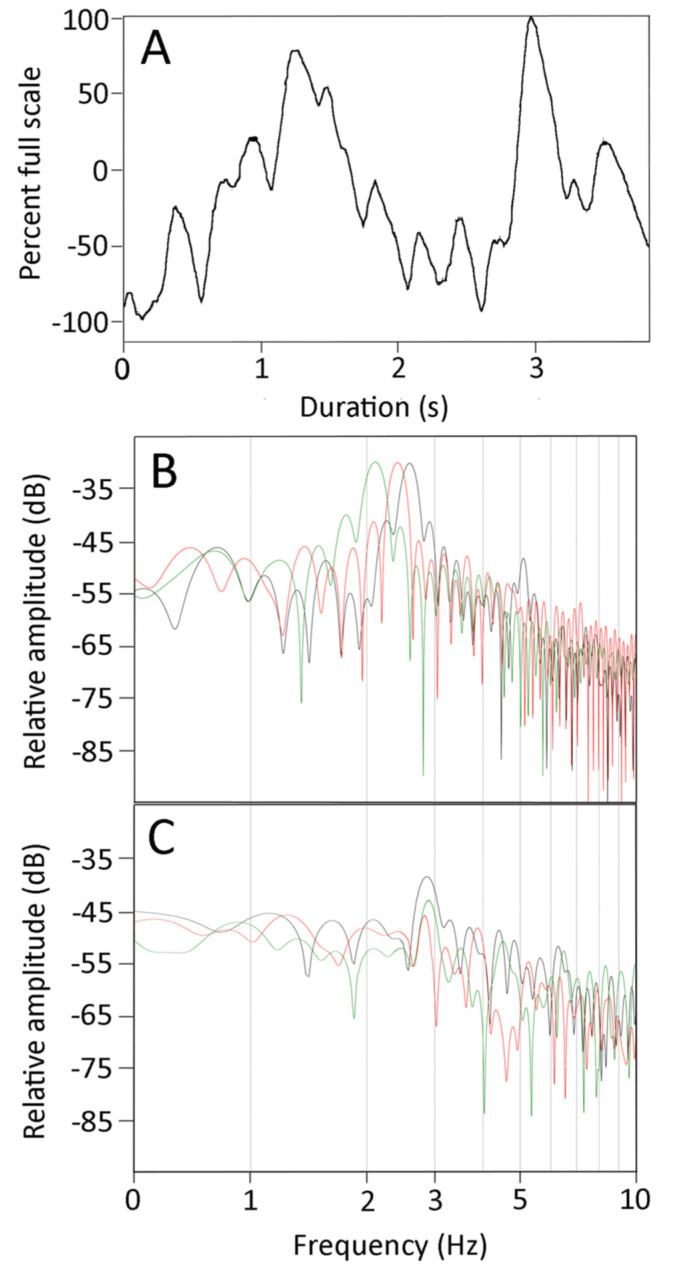
(**A**) Plot of the cranial CSF pressure (Y axis) over time (X axis) during a manual oscillation trial. These pressure traces were characterized by discrete pulses which occurred at frequencies well above those of the ventilatory (0.08 Hz) or cardiac (0.66 Hz) pulses evident in Figure 1A or Figure 3C, and are similar to the 3 Hz frequency of the body oscillations shown in Figure 3B. (**B**) Power spectral analysis of the kinematics from three manual oscillation trials of one specimen. (**C**) Power spectral analysis of the CSF pressure traces from the same three manual oscillation trials. Note the relative similarities of the dominant frequencies (2–3 Hz) and the decrease in spectral power at higher and lower frequencies; the lower frequencies of the CSF pressure are prominent owing to the presence of the cardiac and ventilatory components.

The CSF pressure traces recorded during the manual oscillations were all characterized by the presence of pulsations (Figure 4A). These CSF pulsations, while variable, had frequencies that were much higher than the cardiac or ventilatory frequencies; rather, they occurred over the same frequency range as the kinematic oscillations of the anesthetized snakes (Figure 4A).

FFT and power spectral analyses were performed on the kinematic data of displacement of the snake’s body during the manual oscillations. The results (Figure 4B) show a consistent dominant frequency near 3 Hz (mean for 21 trials was 2.7 Hz, s.e. = 0.06), with a marked drop off in power both below and above the dominant frequency. Power spectral analyses of the CSF pressure tracers recorded synchronously with the kinematics look rather different (Figure 4C). The dominant frequencies were similar (mean for the 21 trials was 3.0 Hz, s.e. = 0.07), but the drop off from the dominant frequency (particularly on the lower side) was much less in the pressure traces. A paired *T*-test revealed that the dominant frequencies from the CSF pressure were significantly higher than those from the kinematic data (*t* = 3.459, *p* = 0.0025, *n* = 21). Regression analysis revealed that increasing the dominant frequency of the kinematic displacement resulted in an increase in the dominant frequency of the CSF traces that was significantly different from zero (*t* = 2.3, *p* = 0.03, df = 19), and that there was considerable variation around the regression line (b = 0.49, R^2^ = 0.22). The kinematic displacement and CSF pressure curves have the same duration. After the curves are rectified and normalized, there is a positive regression relationship (b = 0.495, R^2^ = 0.76) between the areas under the curves which is significantly (*t* = 7.79, *p* = 2.5 × 10^−7^, df = 19) greater than zero (so increasing kinematic displacement increases CSF pressure). 

As shown in Figure 4A, the elevated CSF pressure was not constant, but rather exhibited a series of high-frequency pulsations. Pooled across all of the trials, these pulsations had a mean amplitude of 2.5 mmHg (s.e. = 0.12), which is roughly double the amplitude of the pulsations associated with the cardiac cycle. The amplitude of the CSF pulsations recorded during the manual oscillations were compared to the kinematic variables. Only the propagation velocity of the snake’s undulatory waves had a significant relationship with CSF pulse amplitude; increasing propagation velocity resulted in greater amplitude of the CSF pulsations (b = 0.012, R^2^ = 0.37; this slope was significantly different from zero, *t* = 3.35, *p* = 0.003, df = 19).

### 3.4. Observations on Freely Locomoting Snakes

**Figure 5:**
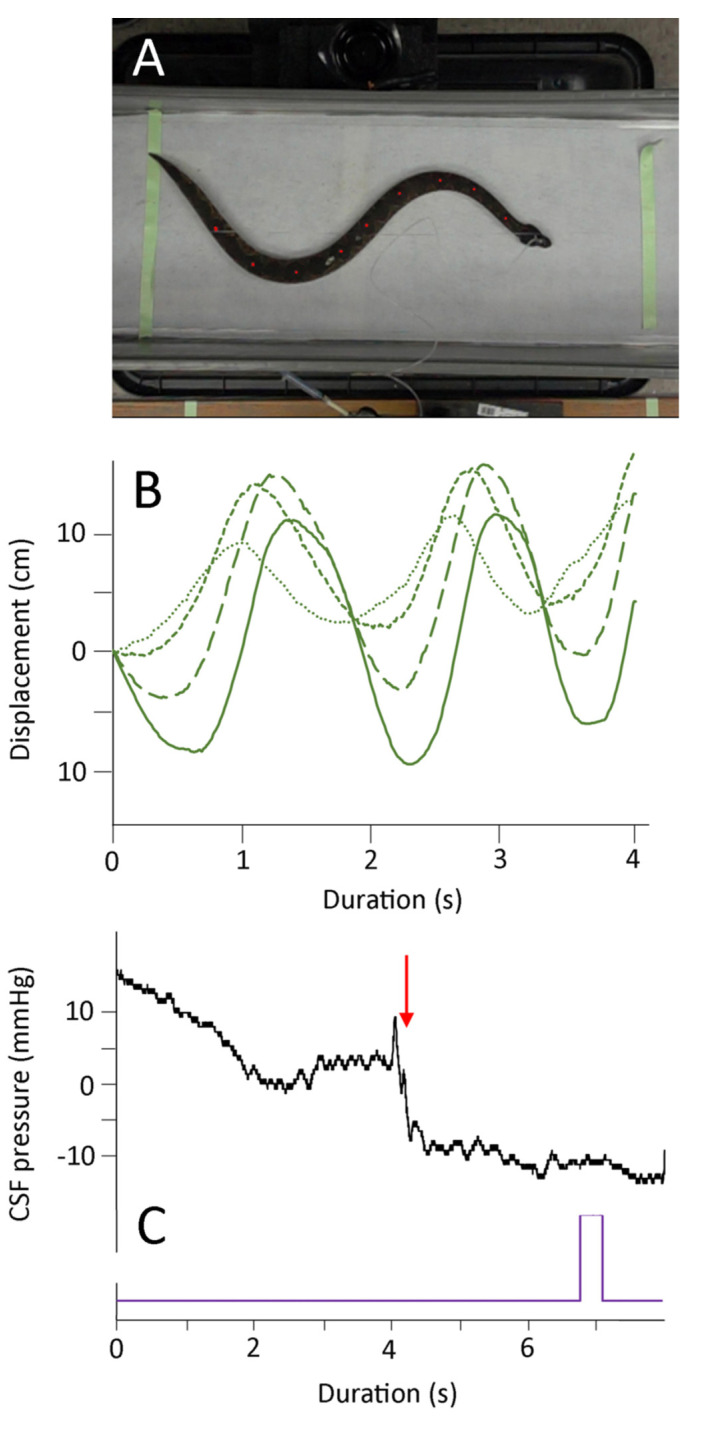
Summary of the undulatory locomotion. (**A**) Isolated frame from the high-speed digital video; note that the snake’s body is not elevated and the marker points on the snake’s dorsal surface are clearly evident. (**B**) Kinematic data showing the lateral displacement of four successive body marker points (successive points have finer line dashes); note that these oscillations occur at a lower frequency than those recorded during manual oscillations (Figure 3B). (**C**) Cranial CSF pressure recorded during an undulatory locomotion trial. The onset of locomotion is marked by a red arrow; the purple marker is an integration signal that appeared in all kinematic and pressure records, allowing for the temporal alignment of the data sets.

As soon as the snake appeared to be fully recovered, it was placed into a filming cage. The pressure catheter, which was still attached to the snake’s skull, was draped over a central support wire in an attempt to reduce signal noise and provide some structural support. The intention was to record CSF pressure from freely undulating snakes. While this worked (Figure 5), it was the exception, not the rule. Only 10 locomotor trials were used for full analysis; five trials involved lateral undulation, and the other five involved concertina locomotion. Concertina locomotion is a specialized mode of locomotion that does not involve the propagation of a body wave (Table 1). ANOVA of the maximum displacements found significant differences among the different kinematics (F = 32.38, *p* = 5.21 × 10^−8^, *n* = 31); Tukey’s post hoc analysis found significant differences (using a threshold of *p* = 0.05) among all three kinematic categories (manual oscillations, lateral undulation, and concertina). ANOVA of the maximum velocities found significant differences among the different kinematics (F = 19.00, *p* = 6.11 × 10^−6^, *n* = 31); Tukey’s post hoc analysis found that concertina locomotion was significantly slower than the other two modes, which were not significantly different. A *t*-test found that the lateral undulation had significantly (*t* = 7.35464, *p* = <0.00001, *n* = 26) slower propagation velocity than the manual oscillations.

**Figure 6:**
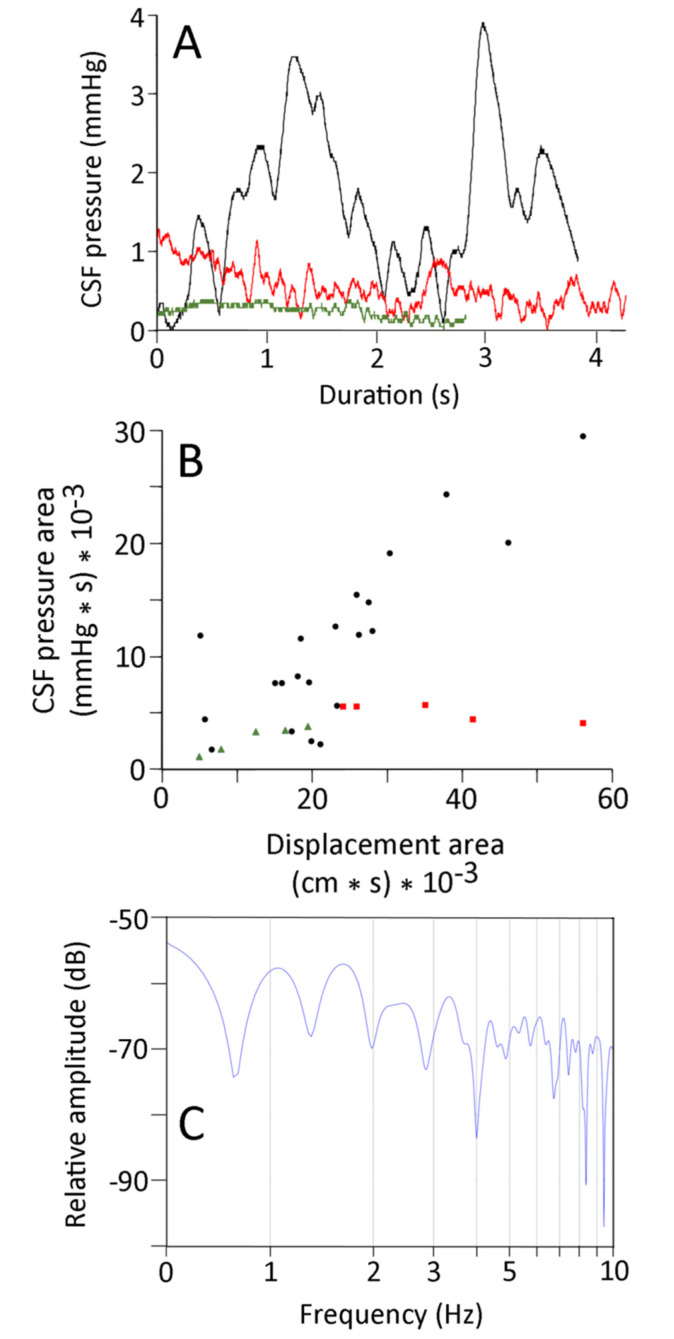
(**A**) CSF pressure traces recorded from the same specimen of *Candoia* during manual oscillations (black trace), undulatory locomotion (red trace), and concertina locomotion (green trace). The traces have not been adjusted to equal lengths, but are presented with the same Y-axis scale. (**B**) Comparison of the area under the kinematic displacement curve (X-axis) and the area under the CSF pressure curve (Y-axis) for the manual oscillation (black circles), undulatory locomotion (red squares), and concertina locomotion (green triangles). Note that the concertina locomotion trials fall on the low end of both the displacement and CSF pressure scales. (**C**) Power spectral analyses of CSF pressure data from an undulatory locomotor trial; note the relative simplicity of the power spectrum.

Compared to the CSF traces recorded during the manual oscillations, the traces recorded during lateral undulation lacked a clear baseline elevation and had smaller, less frequent CSF pulses (Figure 5A and Figure 6A). No clear CSF “response” was recorded while the snakes were performing concertina locomotion (Figure 6A). These kinematic and CSF patterns are evident if the areas under the kinematic displacement and CSF curves are compared. The lateral undulation data overlap with the displacements recorded during manual oscillations, but have lower CSF area (Figure 6B). The concertina locomotion combined low displacements with low CSF areas (Figure 6B). ANOVA confirms that the area under the CSF traces from the manual oscillations is significantly (F = 4.62, *p* = 0.018, *n* = 31) greater than the areas of the CSF traces recorded during either of the locomotor trials.

Not only were the displacement and CSF traces recorded during natural locomotion of lower magnitude than those recorded during the manual oscillations, they were also “simpler”. Both the displacement and the CSF traces from natural locomotion had fewer peaks than those from manual oscillation. The power spectral analyses of the displacement and CSF traces recorded during locomotion in *Candoia* typically exhibited a single harmonic, with a marked decrease over higher frequencies in the displacement traces (Figure 6C), and little drop-off in the CSF traces. There was little evident congruence between the power spectra of the displacement and CSF data sets from either undulatory locomotion or concertina locomotion.

## 4. Discussion

The EKG traces from *Candoia aspera* (Figure 1A and Figure 2) are very similar to EKG traces previously published from other snakes [45,46]. The experiments with *C. aspera* were conducted at 29–31 °C; the mean heart rate of 40 bpm is similar to what has been reported from other species at this temperature [47]. With the terrestrial *C. aspera* under the influence of isoflurane, no compensatory changes in heart rate were found for head-up or head-down orthostatic gradients. Similar results have been reported from other terrestrial snakes [35]. Armelin et al. [48] noted some of the challenges in the literature of this topic, and provided a pharmacological analysis of the orthostatic response of *Boa constrictor*. For the present study, the important issue is that there was no apparent cardiovascular response to tilting that could have influenced the CSF pressure.

Rotating the snake produced a physical pressure head, the magnitude of which depended on the length of the snake. Similar CSF pressure head effects of rotation have been previously shown in alligators [23,24] and cats [22]. The CSF response to tilting in *C. asprea* includes two important findings. First, there was no obvious compensation for the changes in CSF pressure (Figure 2), presumably reflecting the lack of a cardiovascular response to tilting. Second, the magnitude of change for head-down rotation (mean 32 mmHg) was significantly larger than the magnitude for head-up rotation (mean 17.6 mmHg). In mammals, the pressure head is calculated not between the head and the axis of rotation, but between the head and the heart (+ the hydrostatic indifference point [49]). This is because the mammalian heart can effectively isolate the craniad and caudad blood. Previous work with alligators found that the pressure head was based on the distance between the head and the axis of rotation, not the head and the heart [23,24], reflecting the extreme lability of blood in the alligator. The heart of *C. aspera* is located 29.5% of the snake’s body length caudal to the snout, a value that is similar to other fossorial snake taxa (the heart of the more arboreal *C. carinata* is 26% of the body length caudal from the snout [36]). We hypothesize that there is a functional asymmetry to the lability of the blood in *C. aspera*. This could arise due to the presence of an induced venous valve as has been proposed to passively influence blood lability in other snakes [50], or by collapse of the cranial venous drainage [51].

Previous work on *Alligator mississippiensis* reported CSF pressure pulses that closely resembled the “typical” mammalian pattern [6], with lower-frequency, higher-amplitude pulses associated with the ventilatory cycle combined with higher-frequency, lower-amplitude pulses associated with the cardiac cycle. In *Alligator*, the ventilatory and cardiac cycles had highly variable contributions to the CSF pressure profile, both in terms of temporal pattern and amplitude. Young et al. [6] could not discern if this variability was a more common reptilian feature, or if it reflected the unique cardiovascular and respiratory adaptations of Crocodylians. One of the main motives of this study was to assess the relative variability of these two factors in the CSF pressure pulses in *Candoia*. 

The ventilatory and cardiac cycles are readily identified in the CSF traces recorded from *Candoia aspera* (Figure 1A). The identifications were confirmed by: (1) the temporal congruence among the simultaneously recorded variables (Figure 1A); (2) stopping the ventilator to force the snake into apnea (Figure 1A); and (3) power spectral analysis of the CSF pressure, which identified dominant frequencies (Figure 1B) corresponding to the ventilator (~0.08 Hz) and the heart (~0.66 Hz) rates. Unlike the situation described in *Alligator* [6], in *Candoia* the cardiac and ventilatory cycles were a consistent feature of the CSF traces, with the ventilatory cycle contributing roughly 3× the amplitude of the cardiac cycle.

The stability of the ventilatory influence on CSF pressure in *Candoia* is interesting given that *C. aspera*, like all snakes, lacks a diaphragm. The positive pressure ventilatory system used was adjusted (slightly) for each specimen so that there was just enough tidal volume to cause discernible movement of the body wall. Without a discrete intrathoracic space, and pressure, would expansion of the lung (which fills most of the body cavity [32]) have altered jugular venous pressure enough to influence CSF pressure?

There is good evidence for a functional connection between jugular venous pressure and CSF pressure in mammals [21,52,53], but the causal connection between intrathoracic pressure and CSF pressure is not as robust [54,55]. The fact that the alligator, which has a diaphragm capable of maintaining a discrete “intrathoracic” pressure [19], lacks a clear regular pattern of ventilatory-driven CSF pulsations [6], while *Candoia*, which lacks a diaphragm, has a clear functional coupling between ventilation and CSF pulsations, argues that the functional complex between ventilatory movement and CSF pulsations is not a simple one. Further complicating this is the horizontal posture of both *Alligator* and *Candoia*, which presumably minimizes the collapsing of the jugular vein, as is seen in humans during upright posture [51].

The manual oscillations performed on anesthetized *Candoia aspera* established traveling waves of displacement from the tail to the head (Figure 3). These oscillations were always associated with both an increase in CSF pressure, and the presence of pulsations within the CSF that differed from those related to the ventilatory or cardiac cycles (Figure 3 and Figure 4A). The manual oscillations were performed with minimal to no elevation of the anesthetized snake’s tail, and the CSF data collected are difficult to reconcile with an orthostatic explanation. During manual oscillation, the head (and implanted pressure catheter) was nodal (fixed), which was confirmed during the video analysis. Nevertheless, the CSF pressure traces were more “dynamic” than those recorded during the orthostatic trials, and the CSF pulsations recorded during the manual oscillations had unique pulsations, not enhanced cardiac pulsations as during the orthostatic trials (Figure 4A). Furthermore, the significant relationships found between aspects of CSF pressure and both the area under the displacement curve and the propagation velocity, and the similarity in the dominant frequency between the kinematic and CSF pressure traces (Figure 4B,C), argue against an orthostatic explanation.

In *Candoia*, as in all snakes, the epidural space contains vascular elements but is free of the prominent adipose tissue that characterizes this region in mammals [56]. Snakes are well-known for their vertebral flexibility, and *C. aspera* has a characteristic defensive behavior in which it coils its body into a tight ball [57]. The manual oscillations performed on the anesthetized *C. aspera* produced curved bends in the body (Figure 3), not sharp kinks; these bends had a higher radius of curvature than those formed during the snake’s natural defensive posturing. The nature of these curves, combined with the general “openness” of the epidural space in snakes, makes it difficult to attribute the changes in CSF pressure during manual oscillations to an impingement or compression of the spinal dura sheath. 

Given the seeming ubiquity of a myodural bridge in terrestrial vertebrates [26,58,59], we suspect that this anatomical linkage is present in snakes. With the *C. aspera* anesthetized with isoflurane, there should have been little to no skeletal muscle activation during the manual oscillations. Even if passive displacement of the axial muscles was enough to transmit some force to the dura, the pattern of CSF pressures recorded during the oscillations is nothing like what has been recorded during activation of the myodural bridge [28].

We hypothesize that the travelling axial waves created by manual oscillation of the anesthetized *Candoia* resulted in an impulse acting on the spinal CSF, the result of which was a cranial displacement of the CSF and an associated increase in cranial CSF pressure. This explanation is consistent with the significant relationships between propagation velocity and aspects of the CSF pressure, and the general agreement between the power spectral profiles of the kinematic displacement and the CSF pressure (Figure 4B,C). We would not expect a simple 1:1 relationship between oscillation of the body and pulsation of the cranial CSF, primarily due to pressure reflexion within the skull and the irregular nature of the (manually produced) propulsive waves.

When the *Candoia* recovered from the anesthesia and was placed in the filming arena, the object was to look at the influence of naturally occurring body oscillations, not locomotion. To separate those two, the filming arena was lined with a soft smooth fabric that minimized the frictional contact points that are essential for undulatory locomotion in snakes [60]. When the *Candoia* attempted to perform lateral undulation, the undulatory waves produced were of relatively low velocity (Table 1). Whether this was compensation for the lack of frictional contact points, or a remnant effect of the anesthesia, could not be determined. As is common in snakes, particularly heavy-bodied terrestrial species [44,61], *C. aspera* would abandon lateral undulation in favor of concertina locomotion. 

Surface concertina locomotion does not involve propagating loops or waves of axial deflection [44]; when *C. aspera* performed concertina locomotion, we saw no changes in the CSF pressure or pulsations (Figure 6). The lateral undulations performed by *Candoia* had significantly slower propagation than the manual oscillations; because of this, there was a significant difference in the area under the CSF curves of the two undulatory displacements, even though the areas under their displacement curves were not significantly different (Figure 6). The results of the active locomotor trials support the hypothesis of an impulse-based alteration of CSF pressure in *Candoia*. The exact components of the impulse are uncertain. The propagating wave presumably created a craniad impulse, while the abrupt changes in direction associated with manual oscillation could have displaced the spinal cord within the vertebral canal (against the resisting denticulate ligaments), potentially inducing differential CSF flow on either side of the spinal cord. 

There is an extensive body of work modeling the effect of impulses on the displacement and/or deformation of the human brain [62], and inertial forces are a consistent part of the fluid dynamics approach to modeling the CSF [63,64]. Experimental studies of inertial or fluid impulses on the human CSF circulation are rarely done, though Xu et al. [65] found that head rotations alter cranial CSF flow and attributed at least some of the alteration to “motion inertia.” There is a fascinating body of literature on the impact of hyper-, and particularly hypo-, gravity on human intracranial and intraocular pressure [66,67].

We presume that the prominent CSF response to movement reported herein reflects the artificial nature of this experiment, i.e., manual oscillation of an anesthetized snake. Nevertheless, we do think this study supports the findings of Xu et al. [65] in demonstrating that body movement can influence the CSF flow dynamics. Analyses of reptilian or amphibian locomotion may be particularly beneficial to understanding the significance of impulse on CSF flow, particularly along the spinal cord. Many of these taxa move and live nearly horizontally, so the gravitational vector is perpendicular to the main axis of spinal CSF flow. Furthermore, many reptiles and amphibians use traveling axial waves as a regular part of their locomotion [68,69]. Even those taxa that locomote in other ways may provide some novel insights. How does the CSF and brain [70] of the bullfrog (*Rana catesbeiana*) withstand the repeated impulses associated with saltatory locomotion?

## Figures and Tables

**Table 1:** Summary of the kinematic features during the manual oscillations, lateral undulations, and concertina locomotion; values are presented as mean (s.e.). During concertina locomotion there is no propagation of the body segment. Displacements are all given in cm, velocities in cm/s, and frequency in Hz. Note that for velocity calculations, movement away from the long axis of the snake was treated as positive, and movement toward the snake was treated as negative. The manual oscillation data represent three trials on each of the 7 snakes. A total of 5 lateral undulation and 5 concertina locomotion bouts were quantified; these were recorded from 6 of the *Candoia aspera*, with four specimens contributing one of each type of locomotion.

	Manual	Lateral	Concertina
	Oscillations	Undulation	Locomotion
Number of undulations	7.7 (0.6)	2.0 (0.0)	1.0 (0.0)
Propagation velocity	116.9 (5.9)	22.6 (7.9)	
Mean undulation frequency	2.9 (0.1)	0.6 (0.1)	
Mean displacement	2.6 (0.1)	5.4 (0.6)	1.7 (0.7)
Max displacement	6.9 (0.3)	12.5 (0.9)	4.1 (0.9)
Mean velocity	−0.1 (0.1)	0.6 (1.2)	0.2 (0.4)
Max velocity	102.4 (6.0)	77.2 (3.2)	31.8 (2.0)

## Data Availability

The data are available through reasonable request to the corresponding author.

## Statement of Institutional Review

The housing, care, and use of the live *Camdoia aspera* followed all applicable national guidelines and was approved by the Institutional Animal Care and UseThe housing, care, and use of the live *Camdoia aspera* followed all applicable national guidelines and was approved by the Institutional Animal Care and Use Committee of A.T. Still University (Protocol #219, approved 21 October 2020).
